# Accelerating SCF Orbital Optimization with S‑GEK/RVO: Efficient Subspace Compression and Robust Convergence

**DOI:** 10.1021/acs.jctc.5c01714

**Published:** 2025-12-08

**Authors:** Ignacio Fdez. Galván, Daniel Weßling, Roland Lindh

**Affiliations:** † Department of Chemistry for Life Sciences, 8097 Uppsala University, P.O. Box 576, Uppsala 75123, Sweden; ‡ Department of Chemistry − Ångström, Uppsala University, P.O. Box 523, Uppsala 75120, Sweden; § Department of Chemistry and Biochemistry, Faculty of Mathematics and Natural Sciences Institute for Light and Matter, 14309 University of Cologne, Greinstraße 4−6, Köln 50939, Germany; ∥ Uppsala Center for Computational Chemistry (UC_3_), Uppsala University, P.O. Box 576, Uppsala 75123, Sweden

## Abstract

We present enhancements to the S-GEK/RVO method for self-consistent field (SCF) orbital optimization, aimed at improving computational efficiency and robustness. Building on a gradient-enhanced Kriging surrogate model and restricted-variance optimization, we introduce three key modifications: (i) a cost-effective subspace expansion using r-GDIIS or BFGS displacement predictions, (ii) a systematic undershoot mitigation strategy in flat energy regions, and (iii) rigorous coordinate and gradient transformations consistent with the exponential parametrization of orbital rotations. Benchmarking across an extensive set of molecular systems—including organic molecules, radicals, and transition-metal complexes—demonstrates that the new S-GEK/RVO variants consistently outperform the default (in OpenMolcas) r-GDIIS method in iteration count, convergence reliability, and wall time. These improvements make S-GEK/RVO a competitive alternative for SCF optimization and suggest broader applicability to other orbital optimization and localization problems.

## Graphical abstract

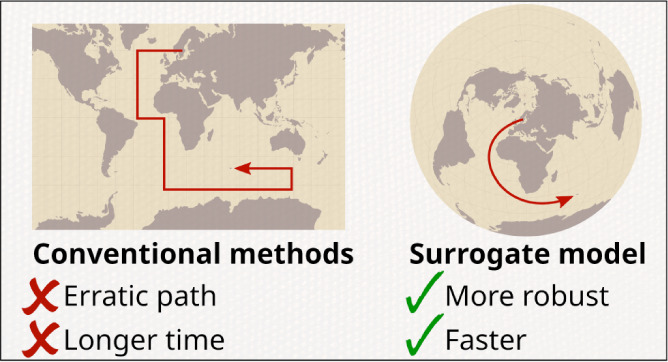

## Introduction

Self-consistent field (SCF) methods are the cornerstone of electronic structure theory, forming the basis of both Hartree–Fock (HF) and Kohn–Sham density functional theory (KS-DFT) calculations. The efficiency and robustness of SCF orbital optimization algorithms have been a central concern since the early days of quantum chemistry. Some prominent methods include a third-order truncated Taylor expansion of the energy with respect to unitary rotations,^1^ the quadratically convergent SCF (QC-SCF) procedure,^2^ and the direct inversion in the iterative subspace (DIIS).^3,4^ This latter method has emerged as one of the most successful, and a number of variations have been more recently suggested.^5–11^ Other recent methods include the augmented Roothan–Hall approach, sometimes used together with DIIS^12–16^ and a number of restricted-step second-order methods^17–22^ as well as hybrid methods aiming at taking advantage of the best features of both DIIS and second-order methods.^23,24^

Although in general all these methods have satisfactory convergence properties, they are not free of problems, and as the electronic structure of the system under study becomes more complex (exhibiting more open-shell, multiconfigurational or near-degenerate character), it is not uncommon to experience poor or failed convergence, resulting in wasted computational resources and sometimes driving the user to the inclusion of ad-hoc constraints with little justification. As illustrated by the continuing publications in the subject, the development of more robust and efficient orbital optimization methods is still an active topic of research.

In a recent work,^25^ two of us proposed a new second-order-like method for SCF optimization, based on a gradient-enhanced Kriging (GEK)^26–28^ surrogate model on a subspace and employing the restricted-variance optimization (RVO), which had already been implemented for geometry optimizations:^29–32^ S-GEK/RVO. The method showed promise in reducing the number of SCF iterations required for convergence and suceeding in all the calculations tested. In spite of these favorable characteristics, its usefulness was limited by the computational cost associated with the second-order procedure used to define the subspace.

In this report, we aim at resolving the issues of S-GEK/RVO and turn it into a competitive and superior method for SCF orbital optimization. Three principal improvements are detailed: the use of a cheap method to define the subspace for the surrogate model to reduce the actual time spent in the calculations, the implementation of a strategy to avoid or mitigate the systematic undershooting in flat surface regions, and the rigorous transformation of coordinates and gradients according to the exponential parametrization to ensure the consistency of the surrogate model.

## Theory

The theoretical framework for SCF orbital optimization has been described multiple times, see e.g., refs 25 and 33–35 and we will not reiterate it here. In this article we compare two basic methods. First, as a reference we use the resetting GDIIS (r-GDIIS) variant developed in ref 25, which is now the default SCF optimization procedure in OpenMolcas, and was shown to outperform the previously established default. Against this baseline, we test two variants of S-GEK/RVO, based on modifications of the method described in ref 25 as well. A short summary of S-GEK/RVO is provided below, for a more detailed presentation we point the reader to refs 25 and 29.

The surrogate model for S-GEK/RVO is expressed as

1 $$E^{*}\left(x\right)=\mu +v(x{)}^{T}M^{-1}y$$

where ***x*** is a vector containing all the independent coordinates, μ is a constant energy value, ***v*** is the generalized covariance vector, that depends on ***x***, ***M*** is the generalized covariance matrix, and ***y*** is the generalized value vector. The latter two do not depend on ***x***, i.e., they are constant for a given surrogate model, but they depend on the data used to build the model. This data is simply a set of energies and gradients computed at previous iterations. The surrogate model is such that it reproduces exactly the data used to build it.

A crucial element of the GEK surrogate model is the definition of the covariance function *f*(***x***, ***x*** ′) that describes the correlation between data points. In practice this function is chosen among some common available ones. Based on previous experience, we choose the so-called Matérn 5/2 function,^36^ defined as

2 $$f\left(x,x^{\prime }\right)=\left(\frac{5}{3}d^{2}+\sqrt{5}d+1\right)\exp\left(-\sqrt{5}d\right)$$

3 $$d=\sqrt{\overset{K}{\underset{k=1}{\sum }}{\left(\frac{x_{k}-x_{k}^{\prime }}{l_{k}}\right)}^{2}}$$

where *K* is the dimensionality of the surrogate model, *x*_*k*_ is the *k*th element of ***x***, and *l*_*k*_ is a constant for each dimension, called the characteristic length. The way to set the *l*_*k*_ values will be discussed later. Qualitatively, *f*(***x***, ***x*** **′**) resembles a *K*-dimensional Gaussian that takes the value 1 when ***x*** = ***x*** **′** and decays to zero when the distance between ***x*** and ***x*** **′** gets larger, at different rates for each dimension, controlled by *l*_*k*_.

Given a set of *m* data points at coordinates {***x***_1_, ***x***_2_, ..., ***x***_*m*_}, the ***M*** matrix is built from all the *f*(***x***_*i*_, ***x***_*j*_) values, as well as all the first and second derivatives (with respect to each dimension of ***x***_*i*_ and/or ***x***_*j*_). The ***v*** vector contains all the *f*(***x***, ***x***_*i*_) and their derivatives with respect to each dimension of ***x***.

The GEK surrogate model also provides a measure for the variance or uncertainty in the energy prediction, which can be estimated as

4 $$s^{2}\left(x\right)=\frac{(y-1\mu {)}^{T}M^{-1}\left(y-1\mu \right)}{m}[1-v\left(x{)}^{T}M^{-1}v\left(x\right)\right]$$

where **1** is a vector that contains 1 at the positions that correspond to energies and 0 at the positions that correspond to gradients. This allows to apply the RVO procedure, where the steps during the optimization are limited not by an arbitrary length, but by the variance predicted from the GEK surrogate model, such that in regions or along directions with a small variance longer steps are allowed.

The characteristic lengths *l*_*k*_ are set noniteratively, in order to reproduce the eigenvalues of a (positive definite) guess Hessian if the model is built with a single data point, which for a Matérn 5/2 covariance function reads:

5 $$l_{k}=\sqrt{\frac{5\left(\mu -E_{\max}\right)}{3\epsilon_{k}}}$$

where *E*_max_ is the maximum energy among the data points, and ϵ_*k*_ is the Hessian eigenvalue corresponding to the *k*th dimension. In our case, we set μ = *E*_max_ + 10*E*_h_.

Dimensionality reduction is achieved by selecting a number of vectors to define a (linear) subspace of the full-dimensional optimization space. The selected vectors are first orthonormalized to generate a set of *K* basis vectors $e_{k}^{f}$ that define the subspace, where the “f” superscript refers to these vectors being written in the full-dimensional space, i.e., their number of components is the total optimization dimensionality. A guess Hessian in the full space can then be projected to the reduced subspace as

6 $$(H^{r}{)}_{ij}=(e_{i}^{f}{)}^{T}H^{f}e_{j}^{f}$$

and diagonalization of ***H*** ^r^ yields a set of basis vectors in the reduced subspace $e_{k}^{r}$ and eigenvalues ϵ_*k*_. The former are used to project the coordinates and gradients from the full space to the subspace. First a projection matrix is defined:

7 $$\mathrm{P̂}=e^{f}e^{r}$$

and then the reduced-subspace coordinates (***x***) and gradients (***g***) are obtained

8 $$x^{r}={\mathrm{P̂}}^{T}x^{f}$$

9 $$g^{r}={\mathrm{P̂}}^{T}g^{f}$$

These ***x*** ^r^ and ***g*** ^r^ are used to build the surrogate model, together with the ϵ_*k*_ to determine the characteristic lengths. The surrogate model is thus completely defined in the reduced subspace. Eventually, only the resulting Δ ***x*** ^r^ obtained in the subspace will need to be back-transformed to the full space, which is done with

10 $$\Delta x^{f}=\mathrm{P̂}\Delta x^{r}$$

The general process for the S-GEK/RVO method is as follows. For each SCF iteration, the energies and gradients from the previous *m* iterations (at most 20) are used to build the surrogate model. First a subspace is defined as the subspace spanned by the *m* displacements and gradients of these data points, plus the current gradient, plus the direction predicted by a second-order RS-RFO procedure, this gives a maximum of 2*m* + 2 orthogonal unit vectors. The guess (diagonal) Hessian is projected onto this subspace and diagonalized, the eigenvectors form the basis in which ***x*** is expressed. From the eigenvalues of this projected Hessian, the *l*_*k*_ values are defined according to eq 5. The coordinates and gradients of the *m* + 1 data points are also projected onto this subspace in order to define the surrogate model according to eq 1. Once this is done, a series of microiterations are performed to find a stationary point on the surrogate model. On this surrogate model, the exact Hessian is available, and we use the RS-RFO method to carry out these microiterations, noting that given the reduced dimensionality and the simple expression of eq 1 the cost of these microiterations is practically negliglible. According to the RVO procedure, the search for the stationary point is limited to a maximum variance (which we set proportional to the gradient): if during the microiterations the variance exceeds the maximum, the step is truncated and the microiterations are interrupted. After a stationary point is found (or the microiterations interrupted), the new point is back-transformed from the model subspace into the full space of orbital rotation coordinates, the orbitals are rotated accordingly, the Fock matrix is recomputed, and a new SCF iteration starts. As usual, the process is repeated until the convergence criteria are met.

We have implemented three specific modifications to this S-GEK/RVO method, as described below.

### Subspace Expansion with Cheap Method

In the S-GEK/RVO method proposed previously,^25^ the full coordinate space for orbital optimization, of dimensionality *n*_occ_ × *n*_vir_, was reduced to 2*m* + 2 (*m* being the number of previous iterations used in the procedure), for the purpose of building the GEK surrogate model. These dimensions were obtained from the displacements and gradients of the previous *m* iterations, as well as the gradient at the current iteration and the displacement predicted by the RS-RFO procedure. It was already identified that this last ingredient was probably the cause for the much degraded performance (with respect to timing, *not* with respect to number of iterations) of the S-GEK/RVO method compared with the r-GDIIS approach.

In this work we replace the use of RS-RFO with other cheaper alternatives. We tested the possibility of using a reduced subspace of only 2*m* + 1 dimensions, defined by the displacements and gradients of the previous *m* iterations and the current gradient, but this did not give satisfactory results. Therefore, we implented additional variants for the subspace expansion by including another vector obtained from different “conventional” displacement predictions. In particular, we have used either the r-GDIIS method (as described in ref 25) or a plain quasi-Newton method with BFGS Hessian update. It is noted that these methods are only used for adding an extra dimension to define the subspace, and therefore only the direction of the predicted displacements, and not their size, are meaningful.

### Systematic Undershoot Mitigation

During our tests, it was found that occasionally the S-GEK/RVO method would converge very slowly. A similar problem was discussed in relation with geometry optimization of weakly bound complexes with RVO.^29^ We suspected this was due to the combination of a very low curvature of the energy surface and a required extrapolation outside the data points used to build the surrogate model. This is illustrated in the left column of Figure 1. The surrogate model is enforced to be bounded, with a positive force constant, which results in an expected systematic undershooting of every optimization step whenever the true surface becomes very flat but still sloped.

**Figure 1:**
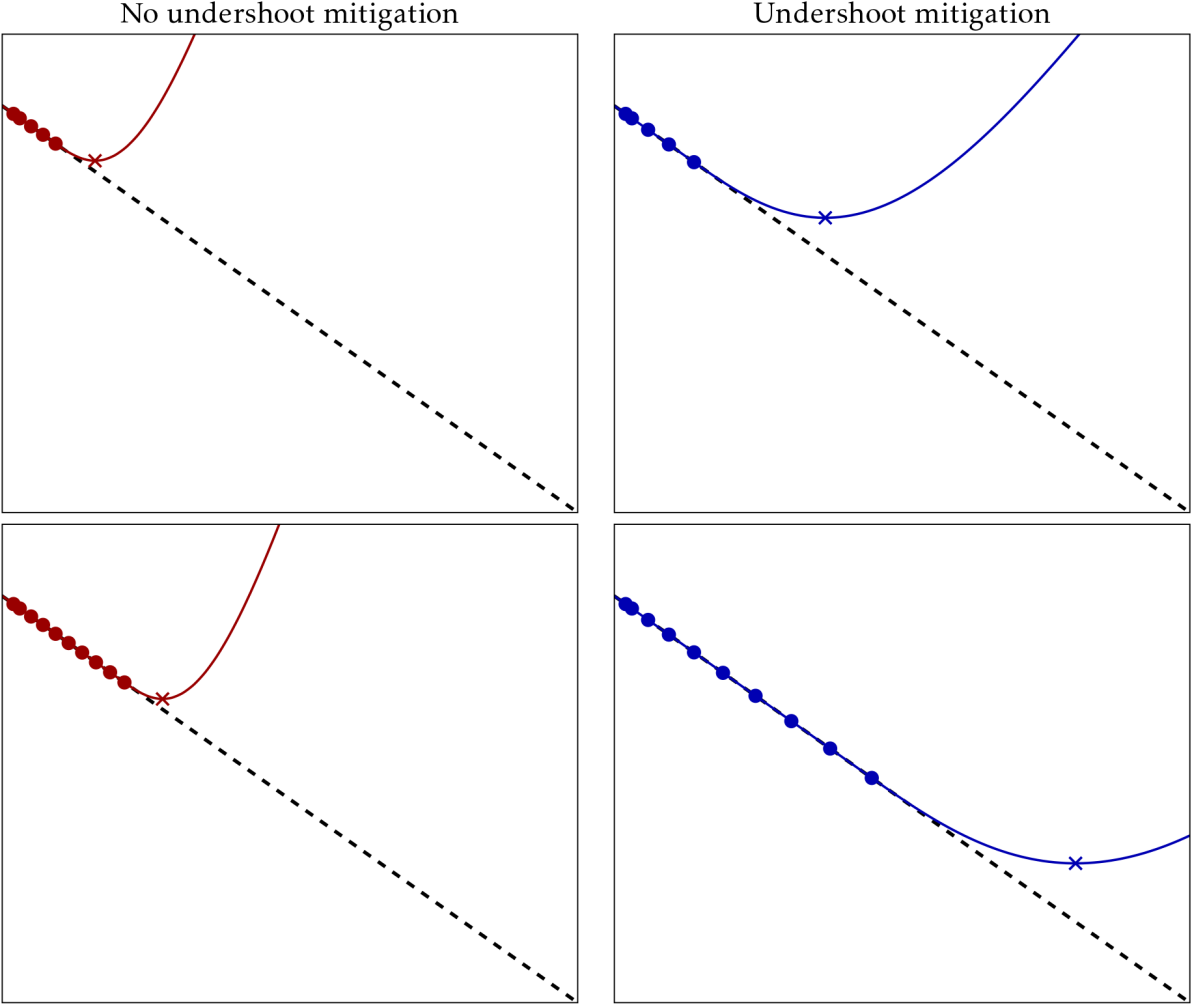
Comparison of the behavior of the RVO method on a one-dimensional straight slope (dashed line) with no specific action to mitigate undershooting (left, red) and with the suggested approach (right, blue). The top row shows the data points (circles), surrogate model (full line) and predicted minimum (cross) after five iterations; the bottom row shows the same after 10 iterations. Note that the point used for the next iteration does not correspond to the predicted minimum because the step size is limited by the maximum variance (not displayed).

Other authors have proposed an overshooting procedure^37^ that could help avoiding such systematic undershooting. We have taken a different approach and, instead of modifying the optimization step, we modify the hyperparameters of the surrogate model, but still take the step yielded by the RVO procedure. In particular, when an undershooting is detected it is probably because the curvature of the actual surface is lower than that of the surrogate model, and therefore we reduce the guessed force constant (increasing the characteristic length) used to build the surrogate model. The effect is displayed in the right column of Figure 1, where it is seen that by reducing the surrogate model curvature the optimization steps become longer, although still limited by the maximum variance, and hopefully escape the flat region faster.

A “loosening factor”, *f*_L_, is defined that starts with the value 1. Then, at every iteration after the first, the resulting step Δ**κ**_*i*_ is compared with the step of the previous iteration Δ**κ**_*i*–1_. If the angle between these vectors is smaller than 5°, *f*_L_ is multiplied by a fixed factor (we use the golden ratio, $\left(1+\sqrt{5}\right)/2)$ . If the angle is larger than 20°, *f*_L_ is reset to 1. In the next iteration, the guessed Hessian matrix, ***H***, is scaled to reduce the force constant along the direction of Δ**κ**_*i*_ according to

$$\begin{array}{cc} W=I+\left(\frac{1}{f_{L}}-1\right)qq^{T} & q=\frac{\Delta \kappa_{i}}{\left|\Delta \kappa_{i}\right|} \end{array}$$ 11

12 $$\overset{∼}{H}=W^{T}HW$$

The scaled matrix ***H̃*** is used to set the characteristic lengths and from the corresponding surrogate model Δ**κ**_*i*–1_ will be obtained.

### Transformation of Coordinates and Gradients

In our previous work,^25^ we employed the common exponential parametrization of the orbital rotations^38,39^ which expresses any relevant orbital rotation matrix as the exponential of an antisymmetric matrix **κ**:

13 $$C^{\prime }=C\exp\left(\kappa \right)$$

where ***C*** is the matrix of coefficients representing a set of molecular orbitals and ***C*** ′ is the corresponding matrix for the rotated orbitals. The Hartree–Fock or Kohn–Sham DFT energy does not change with unitary transformations within the occupied and/or virtual orbitals, only rotations that mix occupied and virtual orbitals are nonredundant. This allows reducing the **κ** matrix to its occupied–virtual blocks, setting the occupied–occupied and virtual–virtual blocks to zero:

14 $$\kappa =\left(\begin{array}{cc} 0 & X\\ -X^{T} & 0 \end{array}\right)$$

where ***X*** is a dense *n*_occ_ × *n*_vir_ matrix whose elements (*X*_*rs*_ = κ_*ia*_) are the independent coordinates modified during the optimization process. It may be the source of some confusion that sometimes **κ** is treated as a vector (e.g., as Δ**κ**_*i*_ in eq 11 above). This should be understood as a simple rearrangement of the elements of ***X*** in a single (column) vector, as a more convenient representation of the full nonredundant variational space of the **κ** matrix in eq 14.

An appealing feature of this parametrization is that the gradient of the energy with respect to **κ** is particularly simple. Indeed, around the null rotation (**κ** = 0) the derivatives are just the elements of the Fock matrix, ***F***, within a constant factor:^35^

15 $${\frac{\partial E(\kappa )}{\partial \kappa_{ia}}|}_{\kappa =0}=-2F_{ia}$$

However, we stress that this identity is only true when evaluated at **κ = 0**. In fact, the expression *E*(**κ**) assumes that a reference set of molecular orbitals ***C*** has been defined, so that other orbitals sets can obtained as a rotation from this reference through eq 13, and the energy is evaluated for this rotated orbital set. More rigorously, it should be written as *E*(**κ;*C***). eq 15 is only valid at the reference orbitals.

When building a surrogate model based on this exponential parametrization, it is important that all the data used for the model be referred to the same set of reference orbitals, as otherwise the data will be inconsistent and the quality of the model will be degraded (if not lead to convergence failures). Whatever choice is taken for the reference orbitals, there will be some transformation needed for the gradients computed according to eq 15 most of the time, and depending on the case coordinates (**κ**) and displacemennts (Δ**κ**) may need to be transformed too.

In the S-GEK/RVO method, we wish to build a surrogate model that accurately represents the *E*(**κ**) surface around the “current” optimization point, which should converge to a minimum. Given that the exponential mapping is most numerically stable around **κ = 0**, we choose to always set the orbitals at the current iteration as the reference orbitals. This means that as the optimization progresses, the reference changes and we must transform the coordinates and gradients of the previous data points to make them consistent with the new reference, while the current gradient can always be computed with eq 15, as it is done at the reference orbitals

### Coordinate Transformation

A given point identified by the coordinates **κ** corresponds, as indicated in eq 13 to a set of orbitals ***C*** ′. When the reference orbitals are changed to a new set ***C̃***, we need to find new coordinates ***κ̃*** that result in the same ***C*** ′ orbitals. The change in the reference orbitals itself comes from a displacement Δ**κ** (***C̃*** = ***C*** exp­(Δ**κ**)), so that we want to find the ***κ̃*** that solves

16 $$C\exp\left(\kappa \right)=\overset{∼}{C}\exp\left(\overset{∼}{\kappa }\right)=C\exp\left(\Delta \kappa \right)\exp\left(\overset{∼}{\kappa }\right)$$

17 $$\exp\left(\overset{∼}{\kappa }\right)=\exp\left(-\Delta \kappa \right)\exp\left(\kappa \right)$$

where the identity exp­(Δ**κ**)^−1^ = exp­(−Δ**κ**) is used. Unfortunately, due to noncommutativity of matrix multiplication, the matrix exponential does *not* follow the rule exp­(***A***)­exp­(***B***) = exp­(***A*** + ***B***) and this *cannot* be further simplified to **κ̃** = **κ** – Δ**κ**, which would be the trivial approach. Moreover, it is found that in general, even when **κ** and Δ**κ** both have the blocked form ([Disp-formula eq14]), **κ̃** will be a dense antisymmetric matrix not complying with that form. But it is always possible to find a **κ̃** of the form ([Disp-formula eq14]) that, while not describing exactly the same set of orbitals ***C*** **′**, describes an equivalent set ***C*** **″** related to ***C*** **′** by redundant rotations among the occupied and virtual orbitals separately, without mixing the two types, and which therefore has the same HF or KS-DFT energy:

$$\overset{∼}{C}\exp\left(\overset{∼}{\kappa }\right)=C^{\prime \prime }=C^{\prime }U^{o,v}$$ 18

19 $$U^{o,v}=\left(\begin{array}{cc} U^{o} & 0\\ 0 & U^{v} \end{array}\right)$$

The nonzero nondiagonal block of such ***κ̃***, ***X̃***, can be found from a singular value decomposition (SVD) of the occupied–occupied block of the product matrix exp­(−Δ**κ**) exp­(**κ**):^38^

20 $$\exp\left(-\Delta \kappa \right)\exp\left(\kappa \right)=\left(\begin{array}{cc} K^{\mathrm{oo}} & K^{\mathrm{ov}}\\ K^{\mathrm{vo}} & K^{\mathrm{vv}} \end{array}\right)$$

$$K^{\mathrm{oo}}=V\Sigma W^{T}\;\left(\mathrm{SVD}\right)$$ 21

22 $$\mathrm{X̃}=-V\mathrm{scos}\left(\Sigma \right)W^{T}(K^{\mathrm{vo}}{)}^{T}$$

where $\mathrm{scos}\left(x\right)=\cos^{-1}\left(x\right)/\sqrt{1-x^{2}}$ and **Σ** is the diagonal matrix of singular values, such that scos­(Σ) is easily computable. The matrix ***U*** ^o,v^ is not needed for coordinate transformation, but it will be necessary for the gradients. It can be obtained as

23 $$U^{o}=VW^{T}$$

24 $$U^{v}=K^{\mathrm{vv}}-K^{\mathrm{vo}}W{\left(\Sigma +I\right)}^{-1}V^{T}K^{\mathrm{ov}}$$

### Gradient Transformation

The gradient at the reference orbitals, eq 15, like the coordinates, can be expressed as blocked antisymmetric matrix

25 $$G\left(0\right)={\frac{\partial E\left(\kappa ;C\right)}{\partial \kappa }|}_{\kappa =0}=\left(\begin{array}{cc} 0 & L\\ -L^{T} & 0 \end{array}\right)$$

and similarly, it can be treated as a vector by rearranging the elements of ***L*** in a column vector. When the reference is changed by Δ**κ**, the same gradient (i.e., the energy derivatives computed at the same orbitals) would correspond to ***G̃*** (−Δ**κ**), which is related to ***G*** (**0**) by^40^

26 $$\begin{array}{ll} \mathrm{G̃}\left(\nu \right)={\frac{\partial E\left(\mathrm{\kappa ̃};\mathrm{C̃}\right)}{\partial \mathrm{\kappa ̃}}|}_{\mathrm{\kappa ̃}=\nu } & =\overset{\infty }{\underset{k=0}{\sum }}\frac{\overset{k\mathrm{times}}{\overset{︷}{[\nu ,\cdot \cdot \cdot [\nu ,}}G\left(0\right)]\cdot \cdot \cdot ]}{\left(k+1\right)!}\\ & =G\left(0\right)+\frac{1}{2}\left[\nu ,G\left(0\right)\right]+\frac{1}{6}\left[\nu ,\left[\nu ,G\left(0\right)\right]\right]+\cdot \cdot \cdot \end{array}$$

In our case, as mentioned above, the orbitals before and after the change of reference are not exactly the same, even though they describe the same wave function and therefore have the same energy. This requires replacing ***G*** (**0**) in eq 26 with ***G*** **″**(**0**):

$$G^{\prime \prime }\left(0;C^{\prime \prime }\right)=(U^{o,v}{)}^{T}G\left(0;C\right)U^{o,v}$$ 27

where the reference orbitals have been made explicit. Taking into account the blocked forms of ***G*** and Δ**κ**, the nonzero block of ***G*** **~,** ***L̃***, can then be expressed directly in terms of the nonzero blocks of ***G*** and Δ**κ**, ***L*** and ***X***, respectively:

28 $$\mathrm{L̃}\left(X\right)=\overset{\infty }{\underset{k=0}{\sum }}\frac{T_{k}}{\left(2k+1\right)!}$$

29 $$P=XX^{T}$$

30 $$T_{0}=(U^{o}{)}^{T}LU^{v}\;\;T_{k}=\left(2XT_{k-1}^{T}-T_{k-1}X^{T}\right)X-PT_{k-1}$$

where all matrix product results as written are of size *n*_occ_ × *n*_vir_ or *n*_occ_ × *n*_occ_, and the series ([Disp-formula eq28]) typically converges in a few terms. We stop the summation whenever the maximum absolute value of the elements of ***T***_*k*_/(2*k*+1)! falls below machine precision.

Alternatively, a noniterative closed formula can be applied based on the eigenvalues and eigenvectors of *i* Δ**κ**,^40^ which is Hermitian and pure imaginary, so it has real eigenvalues and complex eigenvectors. For blocked ***G*** and Δ**κ**, it can be written using only real-valued matrices, in terms of the SVD of ***X*** instead:

31 $$X=R\Gamma Q^{T}\;\left(\mathrm{SVD}\right)$$

32 $$\mathrm{L̃}\left(X\right)=R\left[\left(Z+Z^{T}\right)⊙D^{-}+\left(Z-Z^{T}\right)⊙D^{+}\right]Q^{T}+\left(R\mathrm{sinc}\left(\Gamma \right)R^{T}\right)T_{0}\left(I-QQ^{T}\right)$$

33 $$Z=R^{T}T_{0}Q$$

34 $$D_{ij}^{-}=\frac{\mathrm{sinc}\left(\gamma_{i}-\gamma_{j}\right)}{2}\;\;D_{ij}^{+}=\frac{\mathrm{sinc}\left(\gamma_{i}+\gamma_{j}\right)}{2}$$

where ⊙ denotes an element-wise or Hadamard product, sinc­(*x*)= sin­(*x*)/*x*, and γ_*i*_ are the singular values (diagonal elements of **Γ**). The same ***T***_0_ definition of eq 30 is used.

## Computational Details

We tested the changes described in the previous section on two different benchmark suites. The first one is the same set already used in our previous work,^25^ consisting of 530 organic closed-shell molecules (computed as singlets and triplets), 470 organic radicals (computed as doublets), and 275 transition-metal-containing molecules (computed as singlets and triplets). The second one contains the EXC26 (21 small, well-behaved organic molecules) and MTM (1366 midsize metal–organic molecules) sets of Qin et al.^41^ For a full description of how these suites were designed, please consult the cited works.

All calculations were performed with OpenMolcas^42,43^ version 25.02, tag 88-gd777315f2, which incorporates the methods and modifications here described. SCF optimizations for the organic molecules of the first suite were done at HF level (UHF for doublets and triplets), with the cc-pVDZ basis set.^44–46^ For the systems with transition metals of the first suite, (U)­HF was used as well, with the ANO-R1 (polarized double-ζ) basis set.^47,48^ For the systems in the EXC26 and MTM sets, the SCF optimization was done with DFT, using the functional B3LYP5 (the B3LYP variant that uses VWN5 as the local correlation functional)^49,50^ and the def2-SVP basis set and pseudopotentials^51–54^ as well as the ANO-R2 (polarized triple-ζ) all-electron basis set^47,48^ denoted as EXC26 TZ and MTM TZ. It is noted that the use of ANO-R1 and ANO-R2 basis sets with OpenMolcas automatically enables the exact 2-component (X2C)^55^ scalar relativistic Hamiltonian and finite nuclei. The SCF convergence criteria were the default (energy change below 10^–9^ *E*_h_, a maximum absolute value of occupied–virtual Fock matrix below 1.5 × 10^–4^, and a norm of Δ**κ** below 10^–3^). Two-electron integrals were computed with the RICD approximation,^56^ and in difference with our previous work^25^ the local K (LK) procedure^57^ was employed, as is the default in OpenMolcas. DFT calculations were done with the default Libxc^58^ implementation and numerical quadrature in OpenMolcas. All calculations were conducted without imposing any symmetry constraints. The initial orbitals and preliminary iterations were as described in ref 25 and the “Fermi aufbau” procedure was disabled in all cases. In the following, the number of iterations of the different calculations always exclude these preliminary iterations, which are identical for all methods, except when otherwise stated. The calculations were run on an Intel Core i9-14900K processor, in a “single process” environment, with multithreaded (4 threads) linear algebra library.

## Results and Discussion

For the total 4854 molecular systems in the benchmark set, we report and compare the result of HF or KS-DFT SCF optimizations with three different methods. First, the default OpenMolcas MO optimization scheme, denoted as r-GDIIS, a variant of the DIIS method with quasi-Newton update and a resetting procedure to avoid divergence.^25^ Then with the S-GEK/RVO method as originally described^25^ (S-GEK­(RS-RFO)/RVO), but replacing the use of RS-RFO for subspace expansion with either r-GDIIS (S-GEK­(DIIS)/RVO) or BFGS (S-GEK­(BFGS)/RVO). The systematic undershoot mitigation technique detailed in this work was used for the latter two methods, and the full transformation of coordinates and gradients according to the exponential parametrization was applied to all three methods.

We realized some of the molecules in the benchmark sets are exact or close duplicates. In the MTM set, a total of 17 molecules are exact duplicates, or differ just in atom permutations, of another molecule in the set. In the optimized molecules sets, many geometries are very close (rmsd lower than 10^–3^Å) to some other, but often the differences are enough to cause a change in the convergence pattern with at least one of the methods tested. Still, there are 20 molecules in the singlets and triplets set (they use the same geometries) and 4 in the doublets set that are both practically identical to some other molecule and have indistinguishable SCF convergence behavior with all methods. In all the analyses below we have removed the results of these duplicates (20 from optimized singlets, 4 from optimized doublets, 20 from optimized triplets, 17 from MTM, 17 from MTM TZ).

The summary for the number of SCF iterations necessary to reach convergence is reported in Table 1. For each set we give the number of molecules, the average number of iterations, the standard deviation, the number of cases that took more than 120 iterations, and the number of cases that did not converge within 400 iterations. The statistics for the number of iterations are restricted to the cases that did converge. The latter two figures refer to the total number of iterations, including the preliminary ones. (The number of preliminary iterations is below 10 in 79% of the cases, and is 30 or above in only 23 cases; the maximum is 64.) The results for r-GDIIS and the first 8 sets are roughly similar to those reported in ref 25. The differences are mostly due the different choices regarding the use of the LK algorithm and of the Fermi aufbau procedure, the latter affecting especially the triplet sets. In any case, these differences have no influence on the comparison between the three methods of this work.

**Table 1:** Benchmark Results for the 12 Sets of Benchmark Calculations Compiled with the r-GDIIS, S-GEK­(DIIS)/RVO and S-GEK­(BFGS)/RVO Approaches^a^

	*N*	r-GDIIS	S-GEK(DIIS)/RVO	S-GEK(BFGS)/RVO
singlets	265	11.3 (2.8)/ 0 (0)	10.7 (1.9)/ 0 (0)	10.7 (1.9)/ 0 (0)
singlets opt	245	7.9 (1.1)/ 0 (0)	7.9 (1.0)/ 0 (0)	7.9 (1.0)/ 0 (0)
doublets	235	17.1 (8.1)/ 0 (0)	14.9 (4.7)/ 0 (0)	14.9 (4.9)/ 0 (0)
doublets opt	231	15.2 (13.0)/ 1 (0)	14.0 (10.8)/ 1 (0)	14.0 (11.2)/ 1 (0)
triplets	265	15.9 (8.4)/ 0 (0)	14.4 (5.3)/ 0 (0)	14.4 (5.4)/ 0 (0)
triplets opt	245	25.6 (28.2)/ 5 (1)	18.2 (9.4)/ 0 (0)	18.5 (10.8)/ 0 (0)
TM singlets	275	15.2 (10.4)/ 0 (0)	14.5 (7.3)/ 0 (0)	14.5 (7.3)/ 0 (0)
TM triplets	275	64.6 (47.4)/ 27 (3)	47.2 (25.3)/ 4 (0)	54.6 (39.1)/ 19 (0)
EXC26	21	7.9 (1.4)/ 0 (0)	7.1 (1.0)/ 0 (0)	7.1 (1.0)/ 0 (0)
EXC26 TZ	21	8.3 (1.4)/ 0 (0)	7.4 (1.0)/ 0 (0)	7.4 (1.0)/ 0 (0)
MTM	1349	10.9 (2.8)/ 0 (9)	9.3 (1.7)/ 0 (0)	9.3 (1.8)/ 0 (0)
MTM TZ	1349	11.5 (3.4)/ 0 (10)	9.8 (6.7)/ 1 (0)	9.8 (7.0)/ 1 (0)

a The column *N* gives the total number of molecules in each of the sets. The reported numbers for each of the methods are in the format *a*(*b*)/*c*(*d*), where *a* is the average number of iterations for the calculations that converged, *b* is the corresponding standard deviation, *c* is the number of molecules for which more than a total of 120 iterations was needed, and *d* is the number of molecules which did not converge before a total of 400 SCF iterations.

These results show that both S-GEK/RVO variants have a superior performance (lower average and standard deviation, fewer slow and failed convergences) to the default r-GDIIS, for practically all the sets in the benchmark. Importantly, there is no single convergence failure, whereas r-GDIIS fails in 23 cases. The difference is small but consistent in the benign singlet cases, and especially noticeable in the TM triplets set. It is significant that even when r-GDIIS converges in about a dozen iterations (singlets, EXC26, MTM), the S-GEK/RVO variants can still scratch 0.5 to 1.5 iterations on average. This was already found for S-GEK­(RS-RFO)/RVO,^25^ and confirms that using the alternative methods for subspace expansion does not degrade the behavior. The comparison between S-GEK­(DIIS)/RVO and S-GEK­(BFGS)/RVO is not so clear-cut, the two methods performing much more similarly to each other; it seems, however, that S-GEK­(DIIS)/RVO has in general a slight advantage. We attribute this to the apparent enhanced robustness of DIIS with respect to a plain quasi-Newton method such as BFGS, already reported in ref 25.

To finish the description of these results, we observe that when increasing the basis set size, and therefore the number of orbitals to optimize, from def2-SVP to ANO-R2 (EXC26 and MTM vs EXC TZ and MTM TZ, respectively) the average number of iterations changes very little. This suggests that all three methods are able to capture the essential degrees of freedom relevant to the SCF optimization. To put this into perspective, we recall that the total number of degrees of freedom is *n*_occ_ × *n*_vir_, and that *n*_occ_ does not depend on the basis set size, while *n*_vir_ ranges from 30 to 325 for the def2-SVP calculations and from 86 to 907 for ANO-R2. Thus, the dimensionality of the SCF optimization roughly trebles with the basis set change. On the other hand, all three methods effectively work on a space of much reduced dimensionality, limited to 30 in the case of r-GDIIS and 40 for S-GEK/RVO, independently of the basis set. The number of iterations is on average much smaller than the total dimensionality.

Evaluating performance based on a single statistical measure is problematic,^59^ especially mean and standard deviation, which can be very sensitive to extreme values. One could also argue that it is unfair to exclude nonconverged calculations from the statistics, or to compare calculations that converged to different local minima. For these reasons we perform a more detailed analysis of the results, separating the cases that converge with all methods to the same minimum from those that either do not converge with some method (r-GDIIS) or do so to significantly different minima.

We consider that the calculations converge to the same minimum if the converged energies differ in 10^–8^ *E*_h_ or less and the occupied orbital spaces overlap is 1−10^–6^ or higher. The overlap between orbital spaces is computed as the product of the singular values of $C_{1}^{T}SC_{2}$ ^60,61^ where ***C***_1_ and ***C***_2_ are the MO coefficients of the two orbital sets being compared, and ***S*** is the common AO overlap matrix (as calculations are done with the same basis set and geometry); if the two orbital sets span the same space, the overlap is 1, if at least one of the dimensions of one set is orthogonal to the other set, the overlap is 0. In Figure 2 we compare the distributions for the iteration counts, confined to those cases that converged to the same minimum with all three methods. The EXC26 and EXC26 TZ sets are excluded because the small number of molecules (21) greatly reduces the significance of any statistical measure, even though all of them converged to the same minimum with all methods. As was observed before,^25^ we remark that the singlet calculations, which optimize a restricted Slater determinant, tend to converge in fewer iterations than doublets and triplets, where the determinant is of unrestricted type. This cannot be simply attributed to the larger dimensionality of the optimization space in unrestricted calculations, since the same difference is not found when comparing MTM and MTMZ TZ, where the difference in dimensionality is about 3-fold. Most relevant for the purposes of this work is that the conclusions from Table 1 are maintained in this “fairer” comparison: the S-GEK/RVO variants converge in general in fewer iterations, with fewer extreme cases, except possibly for the optimized singlets set, for which all three methods are extremely efficient and converge almost always in less than 10 iterations. The performance of S-GEK­(DIIS)/RVO and S-GEK­(BFGS)/RVO is roughly the same, only for the transition metal triplets can be said that the former is clearly superior.

**Figure 2:**
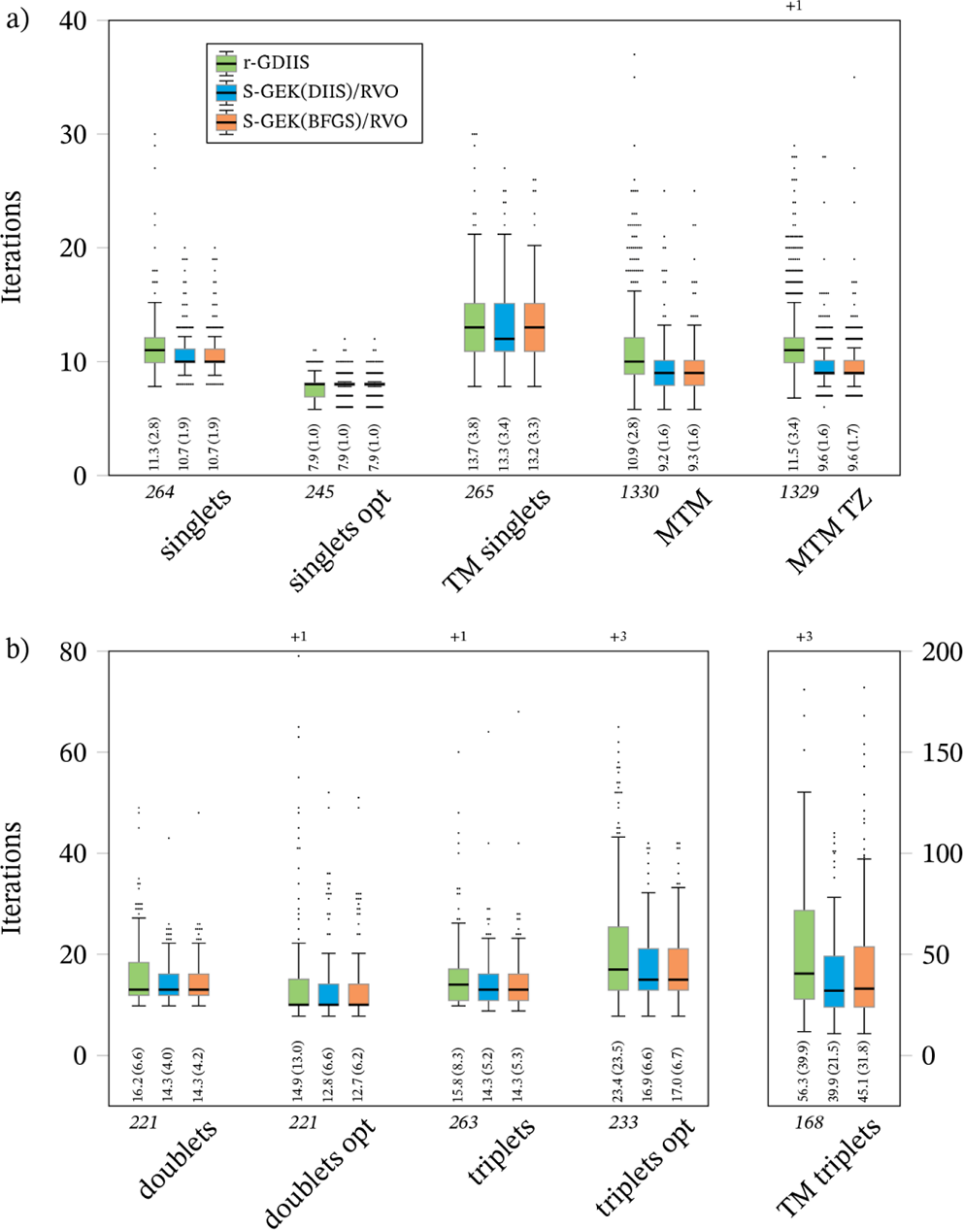
Distributions for the iteration counts of the different benchmark sets, for the cases that converge to the same minimum with all methods: (a) Singlet systems; (b) Doublet and triplet systems. The colored boxes represent range between the first (*Q*_1_) and third (*Q*_3_) quartiles, so they include 50% of the cases. The thick horizontal line marks the median. The whiskers extend at most an additional 1.5 times the interquartile range (IQR = *Q*_3_ – *Q*_1_) beyond the end of the box, but always ending at a data point. Data points outside the range of the whiskers are plotted explicitly, the number of data points outside the graph range are given as a +*n* label at the top of the graph. The numbers in the lower part of the graph (inside) give the mean and, in parentheses, standard deviation. The number in italics below the graph (outside) indicates the number of cases collected in the statistics.

The cases that converged to different minima according to the above criteria, or that did not converge with r-GDIIS, are analyzed in Figure 3. There are a total of 195 molecules affected in this collection, 23 of which did not converge with r-GDIIS. Of the remaining 172, 140 converged to a lower energy with both S-GEK­(DIIS)/RVO and S-GEK­(BGFS)/RVO than with r-GDIIS, and 20 converged to a higher energy with at least one of the two S-GEK/RVO methods (for the other 12, the energy is lower with one method and within the 10^–8^ *E*_h_ threshold with the other). This is visible in Figure 3 as a clear majority of downward-pointing triangles. Regarding the number of iterations, for 100 of the 172 molecules that can be compared both S-GEK/RVO methods converged in the same number of iterations or fewer, within a ±1 threshold, as r-GDIIS; for 55 of them the number of iterations is larger with both S-GEK/RVO methods, and for 17 the behavior of these two methods differs.

**Figure 3:**
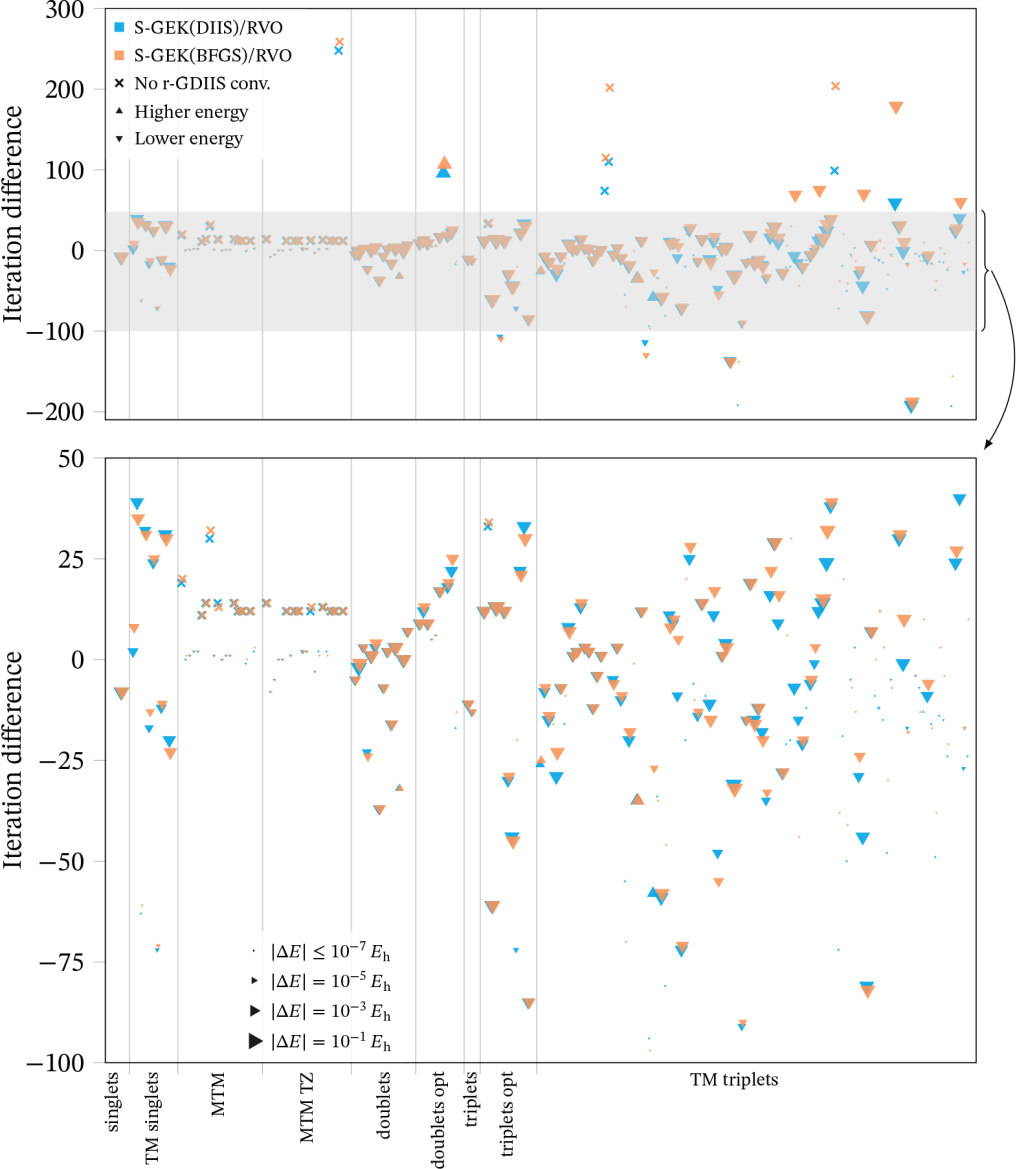
Differences in energy and iteration count with respect to r-GDIIS, for the cases that do not converge to the same minimum with all methods. The calculations that do not converge with r-GDIIS are marked with a cross, and for those the vertical axis is the number of iterations with the S-GEK/RVO method. For the rest, the vertical axis is positive if S-GEK/RVO takes more iterations than r-GDIIS and negative if it takes fewer. The upward-pointing triangles mark calculations that converge to a higher energy with S-GEK/RVO than with r-GDIIS, the downward-pointing triangles converge to a lower energy. The size of the triangle is proportional to the absolute energy difference, according to the legend at the bottom. The horizontal axis is simply a running index, the grid lines divide the different sets, as indicated below the graph. The lower panel is an enlargement of the shaded region in the upper panel.

The three methods compared in this work are local optimization methods that are not guaranteed to find the global minimum, so one should not necessarily take a lower energy as a “better” result. Nevertheless, we suggest that the generally lower energy achieved with S-GEK/RVO is often a desired property. It may be a sign of these methods’ ability to avoid saddle points, where r-GDIIS could be trapped. There is one striking “bad” case for S-GEK/RVO, in the optimized doublets set, for which these two methods take many (about 100) more iterations than r-GDIIS and converge to a significantly higher energy. We identified this as a case where the S-GEK/RVO methods find and explore at what seems a painfully slow rate a very flat region of the energy surface, eventually reaching a minimum, while r-GDIIS quickly skips this region and proceeds to a lower minimum. Indeed, this was the main reason we implemented the systematic undershoot mitigation described earlier, without it the progression is so slow that it does not converge within 400 iterations. We do not rule out that something analogous may occur in the other cases where r-GDIIS converges to a higher energy, often taking more iterations.

As an illustration, we provide Figure 4 displaying the optimization progress for two extreme cases, namely the optimized doublet case just discussed and a TM triplet case where r-GDIIS takes about 140 iterations more than both S-GEK/RVO variants. In the former (top panel), it is seen that r-GDIIS converges relatively smoothly, while the two other methods seem to initially follow a similar path. At around 30 iterations the gradient (represented by the minimum off-diagonal element of the Fock matrix) falls below the convergence threshold and the energy changes are very small, but the displacement is still large and eventually the energy starts to decrease and the gradient fluctuates above and below the threshold. The mauve solid lines at the top show the S-GEK/RVO energies with respect to the r-GDIIS minumum, which is 3.7 × 10^–2^ *E*_h_ lower. Due to the logarithmic scale the details are lost and the line looks almost flat. In comparison, r-GDIIS very soon decreases the energy below that value, probably helped by the “kink” observed at the beginning of the green line. In the TM triplet case (bottom panel), the opposite behavior is observed: now r-GDIIS is stuck at higher energy, 3.3 × 10^–3^ *E*_h_ above the S-GEK/RVO minimum. We note that in the plateau region of the green line the gradient is mostly above the convergence threshold and the energy changes, although small, are somewhat erratic. It takes the method about 100 iterations to get out of this plateau and finally decrease the energy. On the other hand, the S-GEK/RVO methods have a very consistent behavior with monotonically decreasing energy. The mauve line in this case represents the r-GDIIS energy with respect to the S-GEK­(DIIS)/RVO minimum energy, this allows to compare the initial path, which is almost identical for the three methods up to about 15 iterations, where they find a relatively flat region. Both S-GEK/RVO variants soon find a way down in energy, but not r-GDIIS. These, as mentioned, are just two cherry-picked examples, and should not be taken as representative.

**Figure 4:**
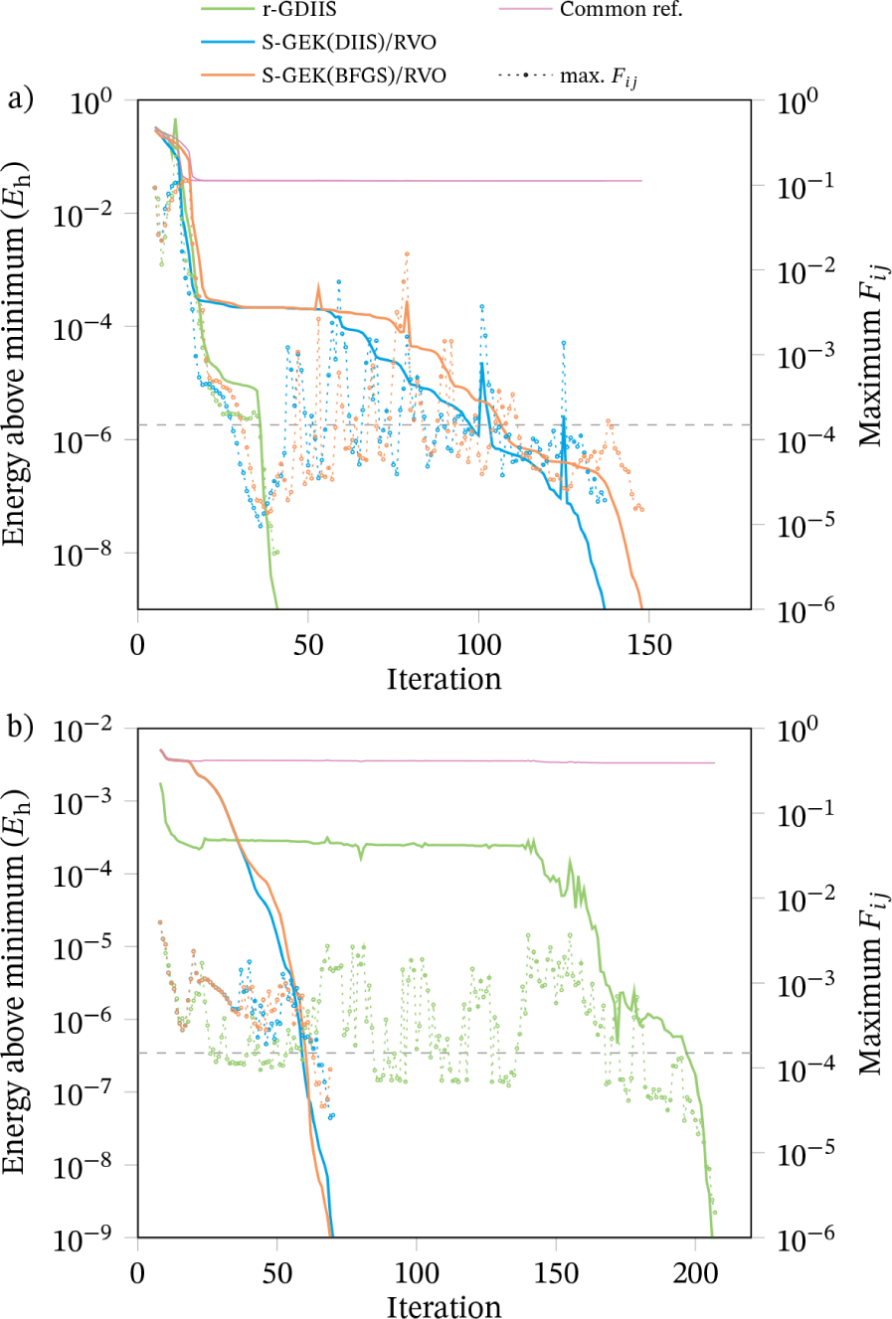
Convergence pattern for two extreme cases: (a) Doublets opt #130, (b) TM triplets #102. Solid lines represent the energy difference with respect to the converged energy of each method (left axis). The mauve lines represent the energy difference with respect to a common reference (the minimum of the three methods). The dotted lines represent the maximum off-diagonal Fock matrix element, as a proxy for the gradient (right axis). The dashed horizontal line marks the convergence threshold for *F*_*ij*_.

Concerning the different sets, it is noticed that most differences are found in the unrestricted calculations (doublets and triplets), especially in the molecules containing transition metals. In all the singlet sets, only 10 cases converge to energy differences above 10^–6^ *E*_h_, the rest (40) are found mostly in the MTM and MTM TZ sets, with either lower energy differences or failed r-GDIIS convergence. Overall, of the 3525 singlet calculations, only 50 (1.4%) show different convergence between the methods, while for the 1251 doublets and triplets the figure is 145 (11.6%).

To finalize the discussion, we draw our attention to the timing of the calculations. The main goal of this work was to improve the performance of S-GEK/RVO with respect to the “wall time” taken by the calculations. The S-GEK­(RS-RFO)/RVO variant previously implemented^25^ already showed a satisfactory behavior in relation to iteration counts, but we noted that the actual time spent was much longer (up to 6 times in some cases) than with r-GDIIS. This was attributed to the RS-RFO subspace expansion step, and is the reason why we implemented the S-GEK­(DIIS)/RVO and S-GEK­(BFGS)/RVO variants, where this costly step is replaced with much cheaper one. It is pleasing to see that this modification had the desired outcome. Table 2 shows the total time spent in the SCF optimization for each set, including the preliminary iterations. Timing comparisons are not straightforward, since no attempt was done to ensure a consistent load and usage of the machine while running the calculations, so the results can only be interpreted qualitatively. It is clear, though, that the S-GEK/RVO variants are competitive timewise, and not only in iteration count, with r-GDIIS: the general reduction of iterations is reflected in a shorter running time by roughly the same proportion. The sometimes large difference in timing between S-GEK­(DIIS)/RVO and S-GEK­(BFGS)/RVO, when the numbers of iterations are almost identical (e.g., the MTM set) is attributed to the intrinsic variability of the timing measurements rather than to a difference beteween the methods. Lastly, it is noted that the time spent in transforming coordinates and gradients, building the S-GEK surrogate model, and performing the microiterations on it, is usually not more than a few seconds, and less than 5% of the total SCF time.

**Table 2:** Total Time Spent in SCF Optimization, in Hours^a^

	r-GDIIS	S-GEK(DIIS)/RVO	S-GEK(BFGS)/RVO
singlets	0.28	0.27 (−3.23%)	0.25 (−8.27%)
singlets opt	0.19	0.19 (+0.14%)	0.18 (−4.78%)
doublets	0.61	0.57 (−6.61%)	0.53 (−11.70%)
doublets opt	0.50	0.48 (−2.90%)	0.45 (−9.19%)
triplets	0.59	0.56 (−5.08%)	0.54 (−8.98%)
triplets opt	0.97	0.72 (−25.45%)	0.69 (−28.21%)
TM singlets	4.55	4.44 (−2.36%)	3.78 (−16.79%)
TM triplets	27.36	20.27 (−25.91%)	21.91 (−19.94%)
EXC26	0.27	0.26 (−4.94%)	0.19 (−27.98%)
EXC26 TZ	6.50	6.19 (−4.88%)	6.14 (−5.53%)
MTM	50.70	44.60 (−12.04%)	33.05 (−34.82%)
MTM TZ	1497.61	1275.60 (−14.82%)	1279.43 (−14.57%)

a In parentheses, the relative difference with respect to the r-GDIIS timing.

## Conclusions

In summary, we have introduced and benchmarked new variants of the S-GEK/RVO method for SCF orbital optimization, replacing the computationally expensive RS-RFO subspace expansion with more efficient alternatives based on r-GDIIS and BFGS displacement predictions. We have also implemented a strategy to mitigate the systematic undershoot that can occur in flat regions of the energy surface, as well as proper transformation of coordinates and gradients along the optimization to ensure consistency of the S-GEK surrogate model.

Our results, based on a benchmark set of close to 5000 molecular calculations, demonstrate that both S-GEK­(DIIS)/RVO and S-GEK­(BFGS)/RVO consistently outperform the default r-GDIIS approach across a wide range of molecular systems, achieving lower average iteration counts, reduced spread, and remarkably no convergence failure. Notably, these improvements are achieved without compromising the quality of the converged solutions, and the computational time is reduced in proportion to the decrease in iterations. The methods are shown to be scalable with respect to basis set size and system complexity, maintaining efficiency even as the dimensionality of the optimization problem increases. The performance difference between S-GEK­(DIIS)/RVO and S-GEK­(BFGS)/RVO is minor, but the results for the most demanding set (TM triplets) somewhat favor the former.

Overall, the proposed enhancements make S-GEK/RVO a competitive and reliable alternative to traditional methods for SCF optimization. It should be straightforward to apply the same technique to other orbital optimization problems, such as found in MCSCF methods,^62^ or in some orbital localization schemes.^63^

## Supplementary Material





## Notes

Coordinates for all molecules in the benchmark sets; results (iterations, energy, time) for all the calculations; list of duplicates (PDF)
(ZIP)

## References

[1] Yaffe L. G., Goddard W. A. (1976). Orbital optimization in electronic wave functions;
equations for quadratic and cubic convergence of general multiconfiguration
wave functions. Phys. Rev. A.

[2] Bacskay G. B. (1981). A quadratically
convergent Hartree–Fock (QC-SCF) method. Application to closed
shell systems. Chem. Phys..

[3] Pulay P. (1980). Convergence
acceleration of iterative sequences. The case of SCF iteration. Chem. Phys. Lett..

[4] Pulay P. (1982). Improved SCF
convergence acceleration. J. Comput. Chem..

[5] Sellers H. (1991). ADEM-DIOS:
an SCF convergence algorithm for difficult cases. Chem. Phys. Lett..

[6] Sellers H. (1993). The C_2_-DIIS convergence acceleration algorithm. Int. J. Quantum Chem..

[7] Kudin K. N., Scuseria G. E., Cancès E. (2002). A black-box self-consistent field
convergence algorithm: One step closer. J. Chem.
Phys..

[8] Garza A. J., Scuseria G. E. (2012). Comparison of self-consistent
field convergence acceleration
techniques. J. Chem. Phys..

[9] Pratapa P. P., Suryanarayana P. (2015). Restarted Pulay mixing for efficient and robust acceleration
of fixed-point iterations. Chem. Phys. Lett..

[10] Li H., Yaron D. J. (2016). A Least-Squares
Commutator in the Iterative Subspace
Method for Accelerating Self-Consistent Field Convergence. J. Chem. Theory Comput..

[11] Hu W., Lin L., Yang C. (2017). Projected Commutator DIIS Method
for Accelerating Hybrid
Functional Electronic Structure Calculations. J. Chem. Theory Comput..

[12] Høst S., Olsen J., Jansík B., Thøgersen L., Jørgensen P., Helgaker T. (2008). The augmented Roothaan–Hall
method for optimizing Hartree–Fock and Kohn–Sham density
matrices. J. Chem. Phys..

[13] Jansík B., Høst S., Johansson M. P., Olsen J., Jørgensen P., Helgaker T. (2009). A stepwise atomic, valence-molecular, and full-molecular
optimisation of the Hartree–Fock/Kohn–Sham energy. Phys. Chem. Chem. Phys..

[14] Wang Y. A., Yam C. Y., Chen Y. K., Chen G. (2011). Communication: Linear-expansion
shooting techniques for accelerating self-consistent field convergence. J. Chem. Phys..

[15] Chen Y. K., Wang Y. A. (2011). LISTb: a Better
Direct Approach to LIST. J. Chem. Theory Comput..

[16] Feldmann R., Baiardi A., Reiher M. (2023). Second-Order
Self-Consistent Field
Algorithms: From Classical to Quantum Nuclei. J. Chem. Theory Comput..

[17] Thøgersen L., Olsen J., Yeager D., Jørgensen P., Sałek P., Helgaker T. (2004). The trust-region self-consistent
field method: Towards a black-box optimization in Hartree–Fock
and Kohn–Sham theories. J. Chem. Phys..

[18] Thøgersen L., Olsen J., Köhn A., Jørgensen P., Sałek P., Helgaker T. (2005). The trust-region self-consistent
field method in Kohn–Sham density-functional theory. J. Chem. Phys..

[19] Yang C., Meza J. C., Wang L.-W. (2007). A Trust
Region Direct Constrained
Minimization Algorithm for the Kohn–Sham Equation. SIAM J. Sci. Comput..

[20] Wen Z., Milzarek A., Ulbrich M., Zhang H. (2013). Adaptive Regularized
Self-Consistent Field Iteration with Exact Hessian for Electronic
Structure Calculation. SIAM J. Sci. Comput..

[21] Helmich-Paris B. (2021). A trust-region
augmented Hessian implementation for restricted and unrestricted Hartree–Fock
and Kohn–Sham methods. J. Chem. Phys..

[22] Kreplin D. A., Werner H.-J. (2022). A combined first-
and second-order optimization method
for improving convergence of Hartree–Fock and Kohn–Sham
calculations. J. Chem. Phys..

[23] Høyvik I.-M. (2020). Convergence
acceleration for the multilevel Hartree–Fock model. Mol. Phys..

[24] Seidl C., Barca G. M. J. (2022). Q-Next: A Fast, Parallel, and Diagonalization-Free
Alternative to Direct Inversion of the Iterative Subspace. J. Chem. Theory Comput..

[25] Sethio D., Azzopardi E., Fdez. Galván I., Lindh R. (2024). A Story of Three Levels
of Sophistication in SCF/KS-DFT Orbital Optimization Procedures. J. Phys. Chem. A.

[26] Liu, W. ; Batill, S. Gradient-Enhanced Response Surface Approximations Using Kriging Models. In 9th AIAA/ISSMO Symposium on Multidisciplinary Analysis and Optimization; AIAA, 2002 10.2514/6.2002-5456.

[27] Han Z.-H., Görtz S., Zimmermann R. (2013). Improving variable-fidelity surrogate
modeling via gradient-enhanced kriging and a generalized hybrid bridge
function. Aerosp. Sci. Technol..

[28] Ulaganathan S., Couckuyt I., Ferranti F., Laermans E., Dhaene T. (2015). Performance
study of multi-fidelity gradient enhanced kriging. Struct. Multidiscip. Optim..

[29] Raggi G., Fdez. Galván I., Ritterhoff C. L., Vacher M., Lindh R. (2020). Restricted-Variance
Molecular Geometry Optimization Based on Gradient-Enhanced Kriging. J. Chem. Theory Comput..

[30] Fdez.
Galván I., Raggi G., Lindh R. (2021). Restricted-Variance
Constrained, Reaction Path, and Transition State Molecular Optimizations
Using Gradient-Enhanced Kriging. J. Chem. Theory
Comput..

[31] Lindh, R. ; Fdez. Galván, I. Molecular structure optimizations with Gaussian process regression. In Quantum Chemistry in the Age of Machine Learning; Elsevier, 2023; pp. 391–428 10.1016/b978-0-323-90049-2.00017-2.

[32] Fdez.
Galván I., Lindh R. (2023). Smooth Things Come in Threes: A Diabatic
Surrogate Model for Conical Intersection Optimization. J. Chem. Theory Comput..

[33] Chavy, C. ; Ridard, J. ; Levy, B. Theory of Orbital Optimisation in SCF and MCSCF Calculations. In Strategies and Applications in Quantum Chemistry; Springer Netherlands, 1996; pp. 19–37 10.1007/0-306-46930-8_2.

[34] Echenique P., Alonso J. L. (2007). A mathematical and
computational review of Hartree–Fock
SCF methods in quantum chemistry. Mol. Phys..

[35] Lehtola S., Blockhuys F., Van Alsenoy C. (2020). An Overview of Self-Consistent Field
Calculations Within Finite Basis Sets. Molecules.

[36] Stein, M. L. Interpolation of Spatial Data; Springer: New York, 1999 10.1007/978-1-4612-1494-6.

[37] Denzel A., Kästner J. (2018). Gaussian process regression for geometry optimization. J. Chem. Phys..

[38] Hutter J., Parrinello M., Vogel S. (1994). Exponential transformation of molecular
orbitals. J. Chem. Phys..

[39] Van
Voorhis T., Head-Gordon M. (2002). A geometric approach to direct minimization. Mol. Phys..

[40] Ivanov A. V., Jónsson E. Ö., Vegge T., Jónsson H. (2021). Direct energy
minimization based on exponential transformation in density functional
calculations of finite and extended systems. Comput. Phys. Commun..

[41] Qin L., Wang Z., Suo B. (2024). Efficient and Robust Ab Initio Self-Consistent
Field Acceleration Algorithm Based on a Semiempirical Model Hamiltonian. J. Chem. Theory Comput..

[42] Fdez.
Galván I., Vacher M., Alavi A., Angeli C., Aquilante F., Autschbach J., Bao J. J., Bokarev S. I., Bogdanov N. A., Carlson R. K., Chibotaru L. F., Creutzberg J., Dattani N., Delcey M. G., Dong S. S., Dreuw A., Freitag L., Frutos L. M., Gagliardi L., Gendron F., Giussani A., González L., Grell G., Guo M., Hoyer C. E., Johansson M., Keller S., Knecht S., Kovačević G., Källman E., Li Manni G., Lundberg M., Ma Y., Mai S., Malhado J. P., Malmqvist P. Å., Marquetand P., Mewes S. A., Norell J., Olivucci M., Oppel M., Phung Q. M., Pierloot K., Plasser F., Reiher M., Sand A. M., Schapiro I., Sharma P., Stein C. J., Sørensen L. K., Truhlar D. G., Ugandi M., Ungur L., Valentini A., Vancoillie S., Veryazov V., Weser O., Wesołowski T. A., Widmark P.-O., Wouters S., Zech A., Zobel J. P., Lindh R. (2019). OpenMolcas: From Source Code to Insight. J. Chem. Theory Comput..

[43] Li
Manni G., Fdez. Galván I., Alavi A., Aleotti F., Aquilante F., Autschbach J., Avagliano D., Baiardi A., Bao J. J., Battaglia S., Birnoschi L., Blanco-González A., Bokarev S. I., Broer R., Cacciari R., Calio P. B., Carlson R. K., Carvalho Couto R., Cerdán L., Chibotaru L. F., Chilton N. F., Church J. R., Conti I., Coriani S., Cuéllar-Zuquin J., Daoud R. E., Dattani N., Decleva P., de Graaf C., Delcey M. G., De Vico L., Dobrautz W., Dong S. S., Feng R., Ferré N., Filatov(Gulak) M., Gagliardi L., Garavelli M., González L., Guan Y., Guo M., Hennefarth M. R., Hermes M. R., Hoyer C. E., Huix-Rotllant M., Jaiswal V. K., Kaiser A., Kaliakin D. S., Khamesian M., King D. S., Kochetov V., Krośnicki M., Kumaar A. A., Larsson E. D., Lehtola S., Lepetit M.-B., Lischka H., López Ríos P., Lundberg M., Ma D., Mai S., Marquetand P., Merritt I. C. D., Montorsi F., Mörchen M., Nenov A., Nguyen V. H. A., Nishimoto Y., Oakley M. S., Olivucci M., Oppel M., Padula D., Pandharkar R., Phung Q. M., Plasser F., Raggi G., Rebolini E., Reiher M., Rivalta I., Roca-Sanjuán D., Romig T., Safari A. A., Sánchez-Mansilla A., Sand A. M., Schapiro I., Scott T. R., Segarra-Martí J., Segatta F., Sergentu D.-C., Sharma P., Shepard R., Shu Y., Staab J. K., Straatsma T. P., Sørensen L. K., Tenorio B. N. C., Truhlar D. G., Ungur L., Vacher M., Veryazov V., Voß T. A., Weser O., Wu D., Yang X., Yarkony D., Zhou C., Zobel J. P., Lindh R. (2023). The OpenMolcas Web: A Community-Driven Approach to Advancing Computational
Chemistry. J. Chem. Theory Comput..

[44] Dunning T. H. (1989). Gaussian
basis sets for use in correlated molecular calculations. I. The atoms
boron through neon and hydrogen. J. Chem. Phys..

[45] Woon D. E., Dunning T. H. (1993). Gaussian basis sets
for use in correlated molecular
calculations. III. The atoms aluminum through argon. J. Chem. Phys..

[46] Wilson A. K., Woon D. E., Peterson K. A., Dunning T. H. (1999). Gaussian basis sets
for use in correlated molecular calculations. IX. The atoms gallium
through krypton. J. Chem. Phys..

[47] Zobel J. P., Widmark P.-O., Veryazov V. (2020). The ANO-R
Basis Set. J. Chem. Theory Comput..

[48] Zobel J. P., Widmark P.-O., Veryazov V. (2021). Correction
to “The ANO-R Basis
Set”. J. Chem. Theory Comput..

[49] Stephens P. J., Devlin F. J., Chabalowski C. F., Frisch M. J. (1994). Ab Initio Calculation
of Vibrational Absorption and Circular Dichroism Spectra Using Density
Functional Force Fields. J. Phys. Chem..

[50] Vosko S. H., Wilk L., Nusair M. (1980). Accurate spin-dependent
electron
liquid correlation energies for local spin density calculations: a
critical analysis. Can. J. Phys..

[51] Andrae D., Häußermann U., Dolg M., Stoll H., Preuß H. (1990). Energy-adjusted *ab initio* pseudopotentials
for the second and third row transition elements. Theor. Chim. Acta.

[52] Metz B., Stoll H., Dolg M. (2000). Small-core multiconfiguration-Dirac–Hartree–Fock-adjusted
pseudopotentials for post-d main group elements: Application to PbH
and PbO. J. Chem. Phys..

[53] Peterson K. A., Figgen D., Goll E., Stoll H., Dolg M. (2003). Systematically
convergent basis sets with relativistic pseudopotentials. II. Small-core
pseudopotentials and correlation consistent basis sets for the post-d
group 16–18 elements. J. Chem. Phys..

[54] Weigend F., Ahlrichs R. (2005). Balanced basis sets
of split valence, triple zeta valence
and quadruple zeta valence quality for H to Rn: Design and assessment
of accuracy. Phys. Chem. Chem. Phys..

[55] Kutzelnigg W., Liu W. (2005). Quasirelativistic theory
equivalent to fully relativistic theory. J.
Chem. Phys..

[56] Aquilante F., Gagliardi L., Pedersen T. B., Lindh R. (2009). Atomic Cholesky decompositions:
A route to unbiased auxiliary basis sets for density fitting approximation
with tunable accuracy and efficiency. J. Chem.
Phys..

[57] Aquilante F., Pedersen T. B., Lindh R. (2007). Low-cost evaluation
of the exchange
Fock matrix from Cholesky and density fitting representations of the
electron repulsion integrals. J. Chem. Phys..

[58] Lehtola S., Steigemann C., Oliveira M. J. T., Marques M. A. L. (2018). Recent developments
in libxc – A comprehensive library of functionals
for density functional theory. SoftwareX.

[59] Weymuth T., Reiher M. (2022). The transferability
limits of static benchmarks. Phys. Chem. Chem.
Phys..

[60] Björck Å., Golub G. H. (1973). Numerical
Methods for Computing Angles Between Linear
Subspaces. Math. Comput..

[61] Jiang S. (1996). Angles between
Euclidean subspaces. Geom. Dedic..

[62] Kreplin D. A., Knowles P. J., Werner H.-J. (2019). Second-order
MCSCF optimization revisited.
I. Improved algorithms for fast and robust second-order CASSCF convergence. J. Chem. Phys..

[63] Høyvik I.-M., Jansik B., Jørgensen P. (2012). Trust Region Minimization of Orbital
Localization Functions. J. Chem. Theory Comput..

