# Supplementary material for: Accelerating SCF Orbital Optimization with S‑GEK/RVO: Efficient Subspace Compression and Robust Convergence
Source: J Chem Theory Comput. 2025 Dec 8;21(24):12674–85. doi: 10.1021/acs.jctc.5c01714 (PMC12746458; doi:10.1021/acs.jctc.5c01714)
Supplement: Supplementary file 1 [file ct5c01714_si_001.pdf]

Supporting Information: Accelerating SCF Orbital Optimization with S-GEK/RVO: Efficient Subspace Compression and Robust Convergence

Ignacio Fdez. Galván,*,†,‡ Daniel Weßling,¶ and Roland Lindh*,†,§

†*Department of Chemistry for Life Sciences, Uppsala University, P. O. Box 576, 75123 Uppsala, Sweden*

‡*Department of Chemistry – Ångström, Uppsala University, P. O. Box 523, 75120 Uppsala, Sweden*

¶*Institute for Light and Matter, Department of Chemistry and Biochemistry, Faculty of Mathematics and Natural Sciences, University of Cologne, Greinstraße 4–6, 50939 Köln, Germany*

§*Uppsala Center for Computational Chemistry (UC_3), Uppsala University, P. O. Box 576, 75123 Uppsala, Sweden*

E-mail: ignacio.fernandez@kemi.uu.se; roland.lindh@kemi.uu.se

Contents of the data.zip file:

- Coords

  Coordinates of the molecules in the different sets, in xyz format. The file names correspond to the molecule id.

  - Doublets: Organic molecules with odd number of electrons
  - Doublets_Opt: Organic molecules with odd number of electrons, geometry optimized as a doublet
  - EXC26: Small organic molecules
  - MTM: Mid-size metal–organic molecules
  - Singlets_Triplets: Organic molecules with even number of electrons, used with both singlet and triplet multiplicity
  - Singlets_Triplets_Opt: Organic molecules with even number of electrons, geometry optimized as a singlet, used with both singlet and triplet multiplicity
  - TM_Singlets_Triplets: Molecules containing transition metal atoms, with even number of electrons (total charge indicated in comment line), used with both singlet and triplet multiplicity

- r-GDIIS, S-GEK_DIIS, S-GEK_BFGS

  Convergence data for each set, in csv format. Rows are ordered according to the numerical order of the molecule ids. The columns in each file are:

  - Prelude: Number of preliminary iterations in the SCF startup procedure. Identical for all methods, not included in most statistics.
  - Iterations: Number of SCF iterations with each method until convergence, not including the preliminary iterations. -1 if the calculation did not converge within 400 total iterations.
  - Energy: Final energy at convergence, or after 400 iterations if it did not converge, in hartree.
  - Time: Time spent in the SCF optimization, in seconds

  In each directory, a separate file is found for each set: Doublets.csv, Doublets_Opt.csv, EXC26.csv, EXC26_TZ.csv, MTM.csv, MTM_TZ.csv, Singlets.csv, Singlets_Opt.csv, TM_Singlets.csv, TM_Triplets.csv, Triplets.csv, Triplets_Opt.csv.

Duplicates

The following molecules in the MTM set are identical, except for a possible permutation of the atoms.

molecule id	duplicate of
1517329	1516666
1517330	1516667
4060460	1100828
4074601	4060844
4074602	4060843
4074940	4060948
4074941	4060949
4088748	4086777
4512494	2107569
4512497	2107554
7051438	7009919
7101457	2105874
7101465	2105882
7101873	4101699
7113689	7103951
7204898	7204897
7212332	7212329

The following molecules are are considered duplicates. The rmsd between them is smaller than 10^{-3} Å, the converged energy difference is below 10^{-4} E_h, and the iteration counts are identical for all methods tested.

molecule ids	duplicate of
Doublets_Opt	
048	019
076	018
191, 192	142
Singlets_Triplets_Opt	
063, 077, 140, 225	042
092	079
107	069
131	120
134, 228	053
142, 239, 259	109
174	101
175	163
179	045
189	059
201	008
211	191
220	000
242	117
